# Regulation of cAMP Responsive Element Binding Protein 3-Like 1 (Creb3l1) Expression by Orphan Nuclear Receptor Nr4a1

**DOI:** 10.3389/fnmol.2017.00413

**Published:** 2017-12-12

**Authors:** Michael P. Greenwood, Mingkwan Greenwood, Benjamin T. Gillard, R. Chitra Devi, David Murphy

**Affiliations:** ^1^School of Clinical Sciences, University of Bristol, Bristol, United Kingdom; ^2^Department of Physiology, Faculty of Medicine, University of Malaya, Kuala Lumpur, Malaysia

**Keywords:** vasopressin, cAMP, transcription factors, transcriptional activation, hypothalamus, methylation

## Abstract

Cyclic AMP (cAMP) inducible transcription factor cAMP responsive element binding protein 3 like 1 (Creb3l1) is strongly activated in the hypothalamus in response to hyperosmotic cues such as dehydration (DH). We have recently shown that Creb3l1 expression is upregulated by cAMP pathways *in vitro*, however the exact mechanisms are not known. Here we show that increasing Creb3l1 transcription by raising cAMP levels in mouse pituitary AtT20 cells automatically initiates cleavage of Creb3l1, leading to a greater abundance of the transcriptionally active N-terminal portion. Inhibiting protein synthesis indicated that *de novo* protein synthesis of an intermediary transcription factor was required for Creb3l1 induction. Strategic mining of our microarray data from dehydrated rodent hypothalamus revealed four candidates, reduced to two by analysis of acute hyperosmotic-induced transcriptional activation profiles in the hypothalamus, and one, orphan nuclear receptor Nr4a1, by direct shRNA mediated silencing in AtT20 cells. We show that activation of Creb3l1 transcription by Nr4a1 involves interaction with a single NBRE site in the promoter region. The ability to activate Creb3l1 transcription by this pathway *in vitro* is dictated by the level of methylation of a CpG island within the proximal promoter/5′UTR of this gene. We thus identify a novel cAMP-Nr4a1-Creb3l1 transcriptional pathway in AtT20 cells and also, our evidence would suggest, in the hypothalamus.

## Introduction

Transcription factor cAMP responsive element binding protein 3 like 1 (Creb3l1), also known as old astrocyte specifically-induced substance (OASIS), was first identified in long-term cultured astrocytes and gliotic tissue (Honma et al., 1999). Creb3l1 is one of five members of the Creb3 basic leucine zipper domain transcription factor subfamily (Kondo et al., 2011). The Creb3 subfamily of transcription factors are structurally similar to activating transcription factor 6, a classical endoplasmic reticulum (ER) stress transducer, but are activated by a diverse range of stimuli in a cell-type specific manner (Chan et al., 2011). Upon stimulation, full-length Creb3l1 protein is cleaved by regulated intramembrane proteolysis (RIP), first by site-1-protease (S1P), giving rise to an intermediate cleavage product, followed by site-2-protease (S2P) cleavage to liberate the N-terminal fragment (Murakami et al., 2006). This active form of Creb3l1 enters the nucleus to activate the transcription of target genes (Omori et al., 2002). Whilst it is well accepted that activation of Creb3l1, like other Creb3 family members, requires RIP (Chan et al., 2011), the mechanisms that mediate the physiological regulation of Creb3l1 are less well understood.

One of the well-documented mechanisms of activating Creb3l1 is by ER stress. Inducing ER stress with tunicamycin or thapsigargin has been shown to activate Creb3l1 RIP (Murakami et al., 2006; Vellanki et al., 2010, 2013). Triggering ER stress also enhances Creb3l1 protein stability in cultured cells (Kondo et al., 2012). In C6 glioma cells and cultured astrocytes, early studies showed that Creb3l1 was involved in the unfolded protein response (UPR), identifying ER chaperone protein 78-kDa glucose-regulated protein (Grp78) as a target of Creb3l1 effects (Murakami et al., 2006). That said, a separate study on the involvement of Creb3l1 in the UPR response in pancreatic beta cells showed that the expression of Grp78 and other UPR target genes expression was not affected (Vellanki et al., 2010). Indeed, it is becoming clear that Creb3l1 has more diverse functions than simply ER stress, with reports of an involvement in secretion, hormone synthesis, the formation of the extracellular matrix and cellular proliferation (Fox and Andrew, 2015). This is backed up by recent reports of activation of Creb3l1 *in vitro* by hypoxia, cytokines and progesterone, suggesting that ER stress is but one of many mechanisms through which the Creb3l1 protein can be activated (Chen et al., 2014; Cui et al., 2015; Ahn et al., 2016).

We recently identified Creb3l1 as a transcription factor of the arginine vasopressin (Avp) gene in magnocellular neurones of the paraventricular nucleus (PVN) and supraoptic nucleus (SON) of the hypothalamus (Greenwood et al., 2014). The rise in plasma osmolality that follows dehydration (DH; complete fluid deprivation) or salt loading (SL; obligate consumption of a 2% w/v NaCl drinking diet) strongly upregulates Creb3l1 expression in vasopressinergic magnocellular neurones in the SON and PVN, with a corresponding increase in the protein abundance. In a later study we used this physiological model to investigate Creb3l1 actions on UPR pathways in these neurones (Greenwood et al., 2015b). Whilst physiological stimuli increased expression of some ER stress genes in parallel with Creb3l1, viral-manipulation of endogenous Creb3l1 action by overexpression of a dominant negative mutant of Creb3l1 in the brain did not alter classical UPR output, suggesting that increased expression of Creb3l1 *in vitro* is not associated with the induction of UPR target genes in these neurones.

Whilst many investigators have focused on mechanisms regulating RIP (Omori et al., 2002; Murakami et al., 2009; Denard et al., 2011; Chen et al., 2014), few have sought to uncover the signaling mechanisms regulating Creb3l1 at the transcriptional level. We have recently reported that endogenous Creb3l1 mRNA expression and cleavage of full-length Creb3l1 are both robustly increased by cyclic AMP (cAMP) pathways in pituitary corticotroph AtT20 cells (Greenwood et al., 2015a), hinting that increased Creb3l1 transcription and cleavage go hand in hand. It is known that cAMP levels increase in the SON in response to hyperosmotic stress (Carter and Murphy, 1989) wherein Creb3l1 mRNA abundance and liberation of N-terminal Creb3l1 also increase (Greenwood et al., 2014) resulting in increased Avp transcription, however the involvement of cAMP pathways in upregulating Creb3l1 expression are not understood.

We have addressed these questions in this study by asking, first, what is the major signaling pathway activating Creb3l1 transcription and, second, what gene products are responsible? Our data describes a novel model of Creb3l1 transcriptional activation by orphan nuclear receptor subfamily 4 group A member 1 (Nr4a1, aka TR3, Nur77) that is affected by Creb3l1 promoter methylation status. We have investigated signaling mechanisms regulating Creb3l1 expression using *in vitro* cell cultures and translated our findings to neurones of the rat hypothalamus. This study identifies Nr4a1 as a putative transcriptional regulator of the Creb3l1 gene.

## Materials and Methods

### Animals

Male Sprague-Dawley rats (purchased from Harlan) weighing 250–300 g were used in this study. Rats were maintained under a 14:10 light dark cycle. Animal experiments were performed between 9 am and 2 pm. We used two protocols a chronic and an acute hypertonic stress protocol. To induce chronic hyperosmotic stress, water was removed (DH) for 3 days or replaced by 2% (w/v) NaCl in drinking water for 7 days SL. The control group had access to food and water *ad libitum*. The acute responses were assessed (10 and 30 min, 1, 2, or 4 h) after a single intraperitoneal injection of hypertonic saline (1.5 ml/100 g body weight of 1.5 M NaCl solution). After injection, hypertonic saline rats were placed back in their home cages and water was removed. The reference group (time 0) had access to food and water *ad libitum*. The study was carried out under a Home Office UK licence (PPL 30/3278) held under, and in strict accordance with, the provision of the UK Animals (Scientific Procedures) Act (1986). The protocols were approved by the University of Bristol Animal Welfare and Ethical Review Board.

### Cells and Treatments

Mouse pituitary cell line AtT20/D16v-F2 (Sigma; 94050406), Human Embryonic Kidney cells HEK293T/17 (ATTC CRL-11268), Neuro 2a cells N2a (ATTC CCL-131) and breast cancer cell line Mcf-7 (a kind gift from Dr. Stephen Lolait, University of Bristol) were cultured in DMEM (Sigma; D6546) supplemented with 10% (v/v) heat-inactivated fetal bovine serum (Gibco), 2 mM L-glutamine (Gibco) and 100 unit/ml of penicillin-streptomycin (Gibco). Cells were incubated at 37°C in a humidified incubator with 5% (v/v) CO_2_. For chemical treatments, cells were seeded onto tissue culture plates to 60%–70% confluence. Transfections were performed using Lipofectamine LTX transfection reagent (Thermo Fisher Scientific, Waltham, MA, USA). Chemical treatments were performed at the time points indicated in the figure; 10 μM forskolin (FSK; Sigma), 100 nM dexamethasone (Sigma), 100 μg/ml cycloheximide (CHX; Clontech), 10 μM SB202190 (Sigma), 10 μM H89 (Abcam), 10 μM U0126 (Cell Signaling Technology, Danvers, MA, USA) and 2.5–10 μM 5-Aza-2′deoxycytidine (Sigma). Stock solutions were prepared in DMSO.

To produce knockdown cell lines, cells were transduced with a lentivirus containing shRNAs targeting c-Fos or Nr4a1. The shRNA sequences (mouse c-Fos shRNA GCTGAAGGCAGAACCCTTTGA, Rat Nr4a1 shRNA1 CAAGTACATCTGCCTGGCAAA and shRNA2 GCCAGACTTATGAAGGCCTCT, human Nr4a1 shRNA GCTACACAGGAGAGTTTGACA) were obtained from the RNAi consortium shRNA library. Sense and antisense oligonucleotides for shRNAs were annealed and ligated into lentiviral transfer vector pLKO.1 puro according to manufacturer’s guidelines (pLKO.1 puro was a gift from Bob Weinberg, Addgene plasmid 8453). A non-targeting (NT) shRNA sequence (ATCATGTTAGGCGTACGGACT) was used as a control. Virus particles were produced in HEK293T/17 cells as previously described (Greenwood et al., 2014). Twenty-four hours after transduction, culture media was replaced with fresh media containing puromycin (2 μg/ml, Thermo Fisher Scientific, Waltham, MA, USA). The transduced cells were subcultured in the presence of puromycin for 2 weeks before use in experiments where puromycin was removed from the culture media.

### Quantitative Real-Time PCR Analysis

The protocols from RNA extraction and cDNA synthesis from brain punches and cells has previously been described (Greenwood et al., 2016b). qRT-PCRs were carried out in duplicate using SYBR green (Roche) on an ABI StepOnePlus Sequence Detection System (ABI, Warrington, UK). For relative quantification of gene expression the 2^−ΔΔCT^ method was employed (Livak and Schmittgen, 2001). The internal control gene used for these analyses were the housekeeping gene Rpl19 and Gapdh. Primers for rat Creb3l1 (5′-GAGACCTGGCCAGAGGATAC-3′ and 5′-GTCAGTGAGCAAGAGAACGC-3′), mouse Creb3l1 (5′-ACAAACTGCAGGGGACATCA-3′ and 5′-GAGCTTGGTGGGGATAGGG-3′), human Creb3l1 (5′-GCAGCCTTGTGCTTTGTTCT-3′ and 5′-GGGGGTCTTCCTTCACAGTC-3′), mouse c-Fos (5′-TCCCCAAACTTCGACCATGA-3′ and 5′-GGCTGGGGAATGGTAGTAGG-3′), rat Fos like antigen 1 (Fosl1) (5′-TCCAGGACCCGTACTTGAAC-3′ and 5′-CTGCTGCTGCTACTCTTTCG-3′), rat CCAAT enhancer binding protein beta (Cebpb) (5′-CAAGCTGAGCGACGAGTACA-3′ and 5′- CAGCTGCTCCACCTTCTTCT-3′), rat Nr4a1 (5′-GCACAGCTTGGGTGTTGATGT-3′ and 5′-GAGGCCATGTCGATCAGTGAT-3′), mouse Nr4a1 (5′- GCACAGCTTGGGTGTTGATGT-3′ and 5′-GAGCCCGTGTCGATCAGTGAT-3′), human Nr4a1 (5′-GGCAAGCTCATCTTCTGCTC-3′ and 5′-CAGGGACATCGACAAGCAAG-3′), rat heteronuclear Avp (hnAvp) (5′-GAGGCAAGAGGGCCACATC-3′ and 5′-CTCTCCTAGCCCATGACCCTT-3′), rat mature Avp (5′-TGCCTGCTACTTCCAGAACTGC-3′ and 5′-AGGGGAGACACTGTCTCAGCTC-3′), mouse mature Avp (5′-GAGTGCCACGACGGTTT-3′ and 5′-AGCTGTACCAGCCTTAGC-3′), rat heteronuclear oxytocin (hnOt) (5′-TGAGCAGGAGGGGGCCTAGC-3′ and 5′-TGCAAGAGAAATGGGTCAGTGGC-3′), rat mature Ot (5′-TGCCCCAGTCTTGCTTGCT-3′ and 5′-TCCAGGTCTAGCGCAGCCC-3′), rat Rpl19 (5′-GCGTCTGCAGCCATGAGTA-3′ and 5′-TGGCATTGGCGATTTCGTTG-3′), mouse Gapdh (5′-CAACTCCCACTCTTCCACCT-3′ and 5′-CTTGCTCAGTGTCCTTGCTG-3′) and human Gapdh (5′-AATCCCATCACCATCTTCCA-3′ and 5′-TGGACTCCACGACGTACTCA-3′) were synthesized by Eurofins MWG Operon.

### Western Blotting

The protocols for extraction of proteins from tissues punches and cells for immunoblotting have previously been described (Greenwood M. P. et al., 2015). Primary antibodies used were goat polyclonal anti-Creb3l1 (1:1000; R&D Systems, AF4080), mouse anti-GAPDH (1:10,000; Santa Cruz, sc-32233), rabbit anti-HA tag (1:10,000, Abcam, ab9110) and mouse anti-Flag (1:2000, Sigma, F1804).

### Immunofluorescent Staining

The protocol for collection and staining of rat brain sections and cultured cells has previously been described (Greenwood et al., 2016b). The fluorescent images were captured using a Leica DMRB microscope with Leica DFC340FX camera using LAS software. Confocal images were obtained using a Leica SP5-II confocal laser scanning microscope attached to a Leica DMI 6000 inverted epifluorescence microscope using LAS software. Primary antibodies were as follows; goat polyclonal anti-Creb3l1 (1:500), mouse anti-cAMP antibody (1:1000; Abcam, ab24851), rabbit polyclonal anti-Nr4a1 (1:50; Santa Cruz, sc-7978), rabbit polyclonal anti-c-Fos (1:25,000; Millipore, PC38) and rabbit anti-HA tag antibody (1:10,000). We have previously described the specificity of the Creb3l1 for Western blotting and immunofluorescent staining applications (Greenwood et al., 2014, 2015a, 2016b).

### Luciferase Assay

For luciferase assays, Creb3l1 promoter fragments were cloned into luciferase reporter construct pGL3 basic vector (Promega, Madison, WI, USA). Initially a 3 kb fragment of the rat Creb3l1 promoter was amplified (5′-CTAGCTAGCATCCCACTGCCCCATCCTGT-3′ and CCGCTCGAGTCACTTTCCGGAGTCTGAAACC) from rat liver genomic DNA template and ligated into the KpnI and XhoI sites of pGL3. This promoter construct was extended by 2 kb by amplification (5′-CGGGGTACCTTGAGAGATGAAGCTGAGCGGTGTG-3′ and 5′-TTCTAGTTCCGGTACCTCCTGTGCAGTCT-3′) of a more distal 5′ promoter region and cloned into the pGL3 KpnI site and Creb3l1 promoter KpnI cut site. A series of smaller rat Creb3l1 luciferase reporter constructs were made by restriction enzyme digestion (BamHI, BstEII, SacI, StuI) at sites present in the Creb3l1 promoter, blunted (with exception of StuI) with T4 DNA polymerase and excised from pGL3 with XhoI. The excised fragments were cloned into the SmaI and Xho1 sites of pGL3. Deletion mutants (-NBRE2 and -NBRE3) of the rat Creb3l1 promoter were generated by overlap extension PCR using Phusion High-Fidelity DNA Polymerase (New England BioLabs, Ipswich, MA, USA). The first round of PCRs were performed using primers (-NBRE2 set 1 5′-TGCAAGCAAACAAGGCAGGTTTACTTTGGGCCCACCACCACCCGGGGCCTG-3′ and 5′-TTCTAGTTCCGGTACCTCCTGTGCAGTCT-3′ and set 2 5′-CGGGGTACCTTGAGAGATGAAGCTGAGCGGTGTG-3′ and 5′-CCCAAAGTAAACCTGCCTTGTTTGCTTGCA-3′; -NBRE3 5′-TGCATCCCAGCAAGAGCCTCACTCTTCGCCCAGGTCCCGGACAGACAGAGG-3′ and 5′-TTCTAGTTCCGGTACCTCCTGTGCAGTCT-3′ and set 2 5′-CGGGGTACCTTGAGAGATGAAGCTGAGCGGTGTG-3′ and 5′-GGCGAAGAGTGAGGCTCTTGCTGGGATGCA-3′). The PCR products from the initial PCRs were combined and used as a template for a subsequent PCR using primers 5′-CGGGGTACCTTGAGAGATGAAGCTGAGCGGTGTG-3′ and 5′-TTCTAGTTCCGGTACCTCCTGTGCAGTCT-3′. The site-directed deletions (-NBRE2 and -NBRE3) were ligated into Kpn1 digested 4.9 kb Creb3l1 promoter pGL3 construct.

*In vitro* methylation of the Creb3l1 promoter plasmid DNA was performed using SssI methylase according to the manufacturer’s instructions (New England Biolabs, Ipswich, MA, USA). Control reactions were performed by omitting the enzyme from the reaction mixture.

Luciferase assays were performed as described previously (Greenwood et al., 2014). Plasmid constructs (0.1 μg pGL3-Creb3l1 promoter + 0.4 μg pcDNA3-Nr4a1) for co-transfection studies or 0.5 μg pGL3-Creb3l1 for chemical treatments, together with 1 ng control renilla reporter pRL-CMV (Promega, Madison, WI, USA) vector/well. The pRL-TK renilla reporter plasmid has previously been shown to be activated by Nr4a1 (Shifera and Hardin, 2010). In the present study pRL-SV40 and pRL-TK renilla reporter plasmids were both activated by Nr4a1 overexpression in AtT20 cells so were not suitable control reporter vectors.

### Chromatin Immunoprecipitation (ChIP)

Cells were seeded (5 × 10^6^) in to 10 cm tissue culture plates. The following day cells were transfected with either 10 μg HA-Nr4a1 or 10 μg Nr4a1 and collected 48 h later. ChIP assays were performed using the Zymo-Spin ChIP Kit (Cambridge Bioscience) with the following modifications. Chromatin shearing was performed by sonication (4 cycles of 30 s on ice) using a MSE Soniprep 150 probe sonicator. Immunoprecipitations were performed with 10 μg of sheared chromatin as input and 1 μg of rabbit anti-HA tag antibody (Abcam, ab9110) at 4°C overnight with rotation. The complexes were captured with 15 μl of Dynabeads Protein G (Thermo Fisher Scientific, Waltham, MA, USA). The ChIP enrichment for the mouse Creb3l1 promoter DNA was determined by RT-PCR using primers NBRE2 (5′-CATTCCCCACAAGTTCCTGC-3′ and 5′-TGTTTGCCTGCCTCGTAAAG-3′) and NBRE3 (5′-TGACTCTCCACCTGACCTTC-3′ and 5′- TCAGTGACGCACAGGAAGAA-3′).

### Dual DNA and RNA Extraction from AVP and OT PVN Punch Samples

Frozen brains were sliced into 60 μm coronal sections in a cryostat set at −20°C. When slices approached the PVN, individual sections were mounted on glass slides and stained with 0.1% (w/v) toluidine blue then visualized on a light microscope until neurones of this brain nucleus were visible. A 27 gauge needle was then used to create small tracts lateral to the posterior magnocellular subdivision of the PVN in the frozen brain tissue in the cryostat sample holder. Then brains were sectioned further, mapping each section, until subdivisions of the PVN were visible. A micropunch (Fine Scientific Tools) with an inner diameter of 0.35 mm was used to collect the posterior magnocellular subdivision of the PVN using the 3rd ventricle and needle tracts from the preceding section as reference points in order to improve the punching accuracy from frozen brain slices. A second more medial 0.35 mm punch was collected from the same side of the PVN and placed into a separate tube. The punches were collected bilaterally from three brain slices and dispensed into 0.5 ml tubes (six punches per tube) placed on dry ice within the cryostat. Total RNA and genomic DNA were extracted from each sample as previously described (Greenwood et al., 2016a).

### Bisulfite Conversion and Sequencing

Genomic DNA from AVP and OT PVN punches (whole sample) and cultured cells (200 ng) was bisulfite converted using Zymo EZ DNA Methylation-Gold kit (Cambridge Bioscience) as previously described (Greenwood et al., 2016a). Primers for amplification of bisulfite converted DNA were designed using MethPrimer software; (mouse 5′-TTAGAGAGTTGAGTTAGTTAAGGAAA-3′ and 5′-CAAAAATCTCTCTAAATCTCTTCC-3′; rat 5′-AGGAAGTTTATAGTTTTTAGGATAG-3′ and 5′-AAAAATCTCTCTAAATCTCTTCCTC-3′; human 5′-TTGGAAGGATGGAAATAGTTTT-3′ and 5′-AAACCCCTAACTAACTAACCCAACTA-3′). The converted DNA was amplified using Platinum Taq DNA Polymerase (Thermo Fisher Scientific, Waltham, MA, USA) with the following cycling parameters: 94°C for 2 min followed by 45 cycles of 94°C for 30 s, 50°C for 30 s and 72°C for 2 min. The PCR products were purified using Qiagen’s PCR purification kit and ligated into pGEM-T Easy vector (Promega, Madison, WI, USA). Ten independent clones were sequenced per sample.

### RNA Scope Protocol

Frozen brains were sliced into 16 μm coronal sections in a cryostat. Sections were individually mounted on Superfrost Plus slides (Thermo Fisher Scientific, Waltham, MA, USA) and stored in slide boxes at −80°C. Tissue was fixed in ice-cold 4% paraformaldehyde for 15 min, followed by 2 × 5 min in phosphate buffered saline washes, and dehydrated by passage through a grade series of ethanol washes (50%–100%) for 5 min each. The brain sections were encircled on the slides using an ImmEdge pen (Vector Laboratories) to create a hydrophobic barrier. The tissue was treated with 100 μl RNAscope protease reagent (Advanced Cell Diagnostics, Newark, CA, USA) for 30 min at room temperature and washed 2 × 2 min in water. A multiplex RNAscope assay was performed using the RNAscope Multiplex Fluorescent Reagent Kit (Advanced Cell Diagnostics, 320850) in accordance with the manufacturer’s guidelines. The RNAscope probes were designed and/or purchased from Advanced Cell Diagnostics. The Rn-AVP probe (Cat No PN 401421) was purchased in channel C2 while a 4ZZ probe targeting bases 2–490 of rat Ot was designed and manufactured by Advanced Cell Diagnostics. All incubations were performed in a HybEZ oven (Advanced Cell Diagnostics, Newark, CA, USA).

### Statistical Analysis

Statistical differences between two experimental groups were evaluated using independent-sample unpaired Student’s *t* tests. One-way ANOVA with Tukey’s *post hoc* test were used to determine the difference between more than two samples with only a single influencing factor. Two-way ANOVA with Bonferroni *post hoc* test was used to determine interactions between two independent variables on the dependent variable.

## Results

### The Rate-Limiting Step in the Generation of Active Creb3l1 Is Transcription

**Figure 1:**
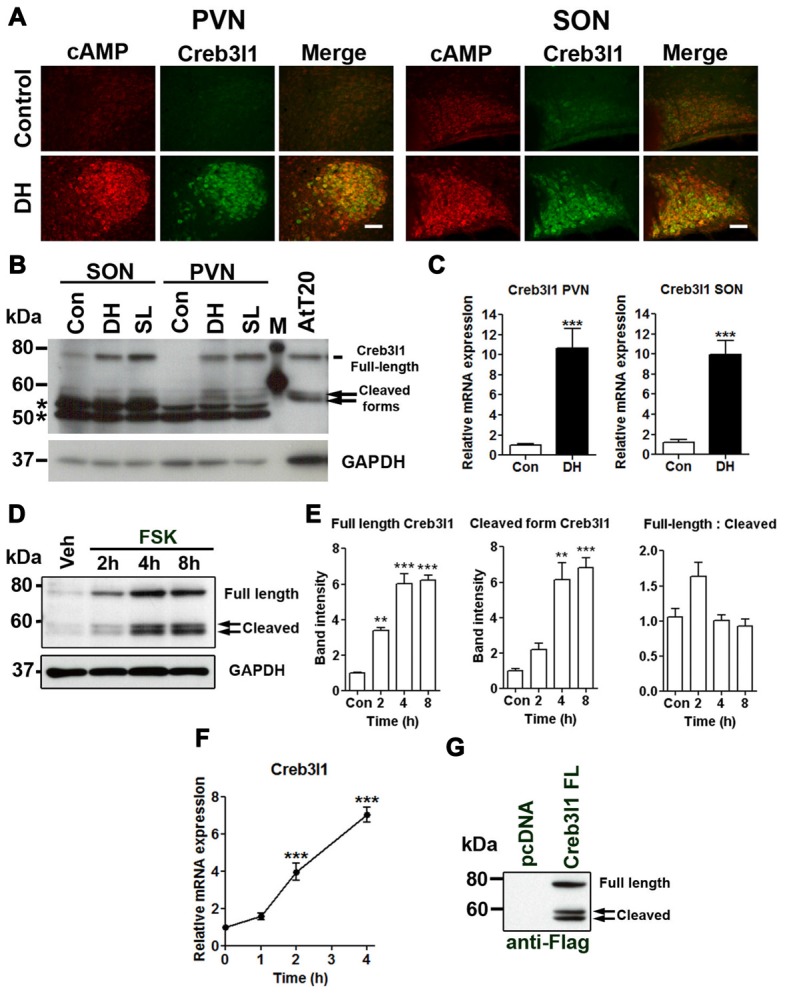
Upregulation of cAMP responsive element binding protein 3-like 1 (Creb3l1) expression by cyclic AMP (cAMP) pathway. **(A)** Immunofluorescent staining in paraventricular nucleus (PVN) and supraoptic nucleus (SON) of euhydrated and 3 days dehydrated rat. **(B)** Immunoblot analysis of total protein extracts from PVN and SON of euhydrated, 3 days dehydrated and 7 days salt loaded rats. **(C)** qRT-PCR analysis of Creb3l1 in PVN and SON of euhydrated and 3 days dehydrated rat. **(D)** Expression of full-length and cleaved Creb3l1 protein in response to forskolin (FSK) treatment. **(E)** Detection of full-length and cleaved Creb3l1 protein forms in AtT20 cells 48 h after transfection with Flag-Creb3l1 construct. Densitometry analysis was performed by using Quantity One imaging software (Bio-Rad). **(F)** Upregulation of Creb3l1 mRNA by foskolin in AtT20 cells determined by qRT-PCR. **(G)** Immunoblot showing Creb3l1 cleavage products in AtT20 cells overexpressing N-terminal Flag tagged Creb3l1 construct. In **(B)** *indicates the detection of non-specific bands by the Creb3l1 antibody in brain samples. DH, dehydration; SL, salt loading; M, molecular weight marker. Values are means + SEM of *n* = 3−4 per group. **p < 0.01, ***p < 0.001. Scale bars = 100 μm.

We have previously shown that Creb3l1 expression is increased by cAMP pathways using *in vitro* cell cultures and in *ex vivo* hypothalamic cultures (Greenwood et al., 2015a). Using a specific antibody, we identified increased cAMP levels in magnocellular neurones of the PVN and SON with osmotic stress, in parallel, and consistent with an increase of Creb3l1 protein expression (Figure 1A). Immunoblot analysis of protein extracts from punches of the PVN and SON revealed an increase in full-length and cleaved forms of Creb3l1 as a consequence of DH and SL (Figure 1B). These changes occur in parallel with an increase in the abundance of Creb3l1 mRNA in both PVN and SON of 3 days dehydrated rats (Figure 1C). To determine any relationship between Creb3l1 expression and restrictions of RIP, we conducted densitometric analysis of full-length and cleaved Creb3l1 bands in FSK treated AtT20 cells (Figure 1D). We found that both full-length and cleaved Creb3l1 protein forms increase by equivalent levels, and the ratio of full-length—cleaved Creb3l1 protein is not significantly altered (Figure 1E). Therefore, the rate of cleavage would appear to increase with increasing Creb3l1 mRNA abundance (Figure 1F). These data argue against there being any mechanism eliciting stimulus dependent regulation of RIP. In agreement, the ratio of full-length Creb3l1 protein to intermediate and active N-terminal forms persists in AtT20 transfected to over-express Creb3l1, despite the high Creb3l1 protein loads placed on RIP system and in the absence of any stimuli (Figure 1G).

### Identification of Transcriptional Regulators of Creb3l1

**Figure 2:**
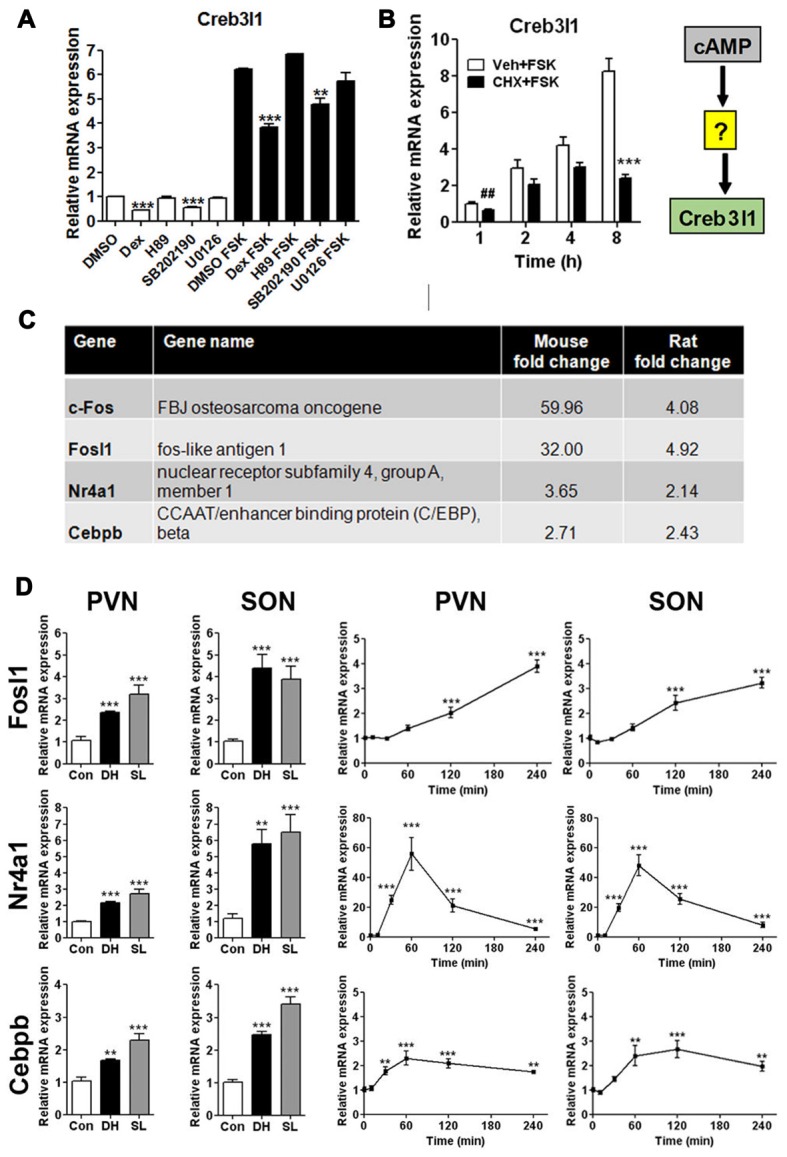
Regulation of Creb3l1 expression. **(A)** AtT20 cells were pretreated with signaling pathway inhibitors for 24 h before treatment with vehicle (DMSO) or stimulation with FSK (10 μM) for 6 h. The expression of Creb3l1 was evaluated by qRT-PCR. **(B)** AtT20 cells were pretreated with CHX or vehicle for 2 h followed by FSK addition to culture media for up to 8 h and Creb3l1 mRNA expression was investigated by qRT-PCR. **(C)** Transcription factors mined from microarray data that increase by greater than two-fold in the SON of the dehydrated mouse (2 days) and rat (3 days) compared to euhydrated controls. **(D)** Relative mRNA expression was investigated by qRT-PCR in PVN and SON of euhydrated, dehydrated (3 days) and salt loaded (7 days) rats and after a single intraperitoneal injection of 1.5 ml/100 g body weight 1.5 M NaCl solution over a 4 h experimental period compared to controls. DH, dehydration; SL, salt loading; CHX, cycloheximide. Values are means + SEM of *n* = 5–6 per group (animal) *n* = 3 per group (cell). ^##^*p* < 0.01 (*t*-test). **p < 0.01, ***p < 0.001.

We treated AtT20 cells with signal pathway specific inhibitors to investigate different routes of Creb3l1 transcriptional activation initiated by cAMP pathways (Figure 2A). Transcript levels of Creb3l1 decreased most markedly following treatment with dexamethasone and p38 inhibitor SB202190 in basal and FSK-stimulated states (Figure 2A). To test if cAMP directly activates Creb3l1 transcription, or if an intermediary step requiring protein synthesis was necessary, we performed studies with the protein synthesis inhibitor CHX. Using cells pretreated with CHX, we asked if cAMP pathways could directly increase Creb3l1 expression (Figure 2B). Inhibition of protein synthesis attenuated FSK-induced Creb3l1 expression, suggesting that *de novo* protein synthesis was necessary for up-regulation of Creb3l1 expression.

We reasoned that the mRNA encoding this intermediate protein must be increased in expression in the SON following DH. We thus mined our previously published microarray data of the dehydrated mouse and rat SON (Hindmarch et al., 2006; Stewart et al., 2011) and, by performing cross-species comparisons, narrowed our list of candidates to four transcription factors (Figure 2C)—c-Fos, Fosl1, Nr4a1 and Cebpb. We have previously described that activation profile of c-Fos and Creb3l1 in the same samples of chronic and acute hyperosmotic stress (Greenwood et al., 2015a). The expression of Fosl1, Nr4a1 and Cebpb were also robustly increased in SON and PVN of the rat by DH and SL, consistent with our array data (Figure 2C). Using our acute hyperosmotic stress protocol we observed markedly different transcriptional activation profiles for these three genes (Figure 2D). We previously showed in these samples that Creb3l1 mRNA increases as early as 1 h after hypertonic saline injection in the hypothalamus (Greenwood et al., 2015a) so allowing for protein synthesis only c-Fos and Nr4a1 were deemed to be viable candidates. These two transcription factors are already well-known to be activated by cAMP pathways (Fass et al., 2003).

### Identification of Nr4a1 as a Novel Regulator of the Creb3l1 Gene

**Figure 3:**
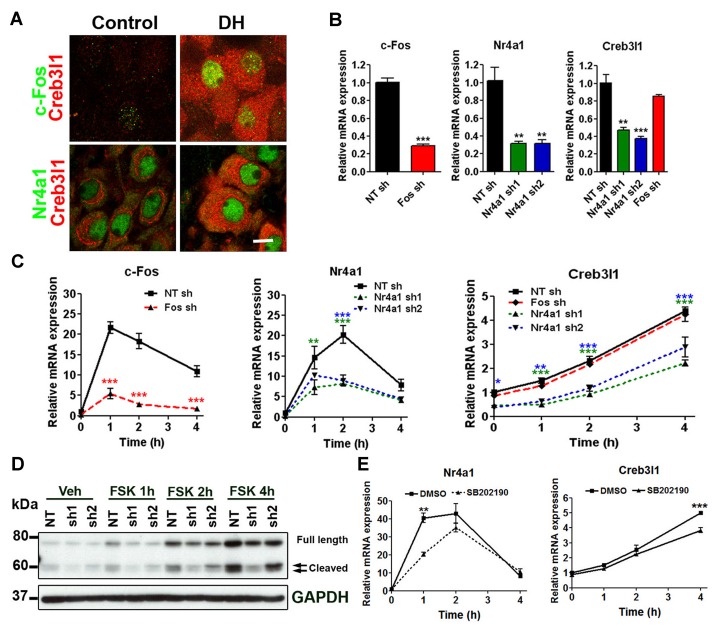
Identification of Nr4a1 as a novel regulator of the Creb3l1 gene. **(A)** Immunofluorescent localization of Creb3l1 with Nr4a1 and c-Fos in magnocellular neurones of euhydrated and 3 days dehydrated rat. **(B)** The effect of c-Fos and Nr4a1 knockdown on basal expression of Creb3l1 were examined by qRT-PCR compared to a control NT shRNA cell line. **(C)** Responses of knockdown cell lines to 4 h time course of FSK treatment compared to a control NT shRNA cell line. **(D)** Immunoblot analysis of Creb3l1 expression in Nr4a1 knockdown cell lines treated with FSK. **(E)** The effect of pretreatment (2 h) of p38 inhibitor SB202190 on FSK-induced upregulation of Nr4a1 and Creb3l1 mRNA in AtT20 cells. NT, non-targeting; FSK, forskolin. Values are means + SEM of *n* = 3 per group. *p < 0.05, **p < 0.01, ***p < 0.001.

Immunofluorescent staining showed that Creb3l1 expressing magnocellular neurones also express c-Fos and Nr4a1, particularly in animals that have been dehydrated for 3 days (Figure 3A). Therefore, we further assessed our two candidates in relation to Creb3l1 expression in our AtT20 cell model. To directly assess any roles of c-Fos and Nr4a1 on Creb3l1 expression, we prepared knock down cell-lines of c-Fos and Nr4a1 in AtT20 using lentiviral vectors, and assessed basal (Figure 3B) and FSK stimulated expression levels (Figure 3C). Knockdown of Nr4a1 with two separate shRNAs significantly reduced basal and stimulated Creb3l1 mRNA and protein expression, whereas knockdown of c-Fos did not (Figures 3B–D). Treatment of AtT20 cells with p38 inhibitor also decreased FSK upregulation of Nr4a1 and Creb3l1 mRNA expression, consistent with our earlier data suggesting that this pathway regulates Creb3l1 expression (Figure 3E).

### Nr4a1 Regulates Creb3l1 Transcription by Binding at the Promoter

**Figure 4:**
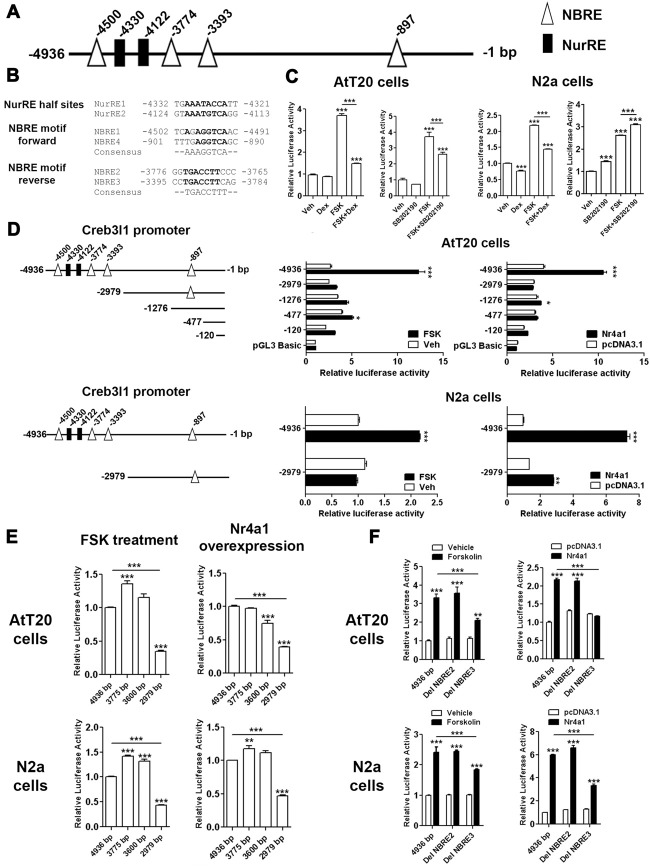
Identification of Nr4a1 as a novel regulator of the Creb3l1 gene. **(A)** Schematic representation of potential Nr4a1 responsive elements in the rat Creb3l1 promoter region. **(B)** Sequences of NurRE and NBRE sites in the rat Creb3l1 promoter. **(C)** Rat Creb3l1 promoter-luciferase reporter constructs with chemical treatments (4 h) in AtT20 and N2a cells. **(D,E)** Luciferase assays using plasmids containing deletion constructs of the Creb3l1 promoter-luciferase reporter together with overexpression of Nr4a1 or 4 h FSK treatment. **(E)** Restriction enzyme generated deletion mutants of the Creb3l1 promoter to pinpoint the interacting site of Nr4a1 with the promoter. **(F)** Luciferase assays using Nr4a1 responsive element site-directed deletion constructs of the Creb3l1 promoter with overexpression of Nr4a1 or 4 h FSK treatment. Veh, vehicle; FSK, forskolin; DH, dehydration. Values are means + SEM of *n* = 3−4 per group. *p < 0.05, **p < 0.01, ***p < 0.001.

To investigate Nr4a1 actions on the Creb3l1 promoter, luciferase reporter assays were performed in AtT20 cells and neuronal cell line N2a. Sequence analysis of 4.9 kb segment of DNA upstream of the transcriptional start site of the rat Creb3l1 gene revealed potential Nr4a1 binding motifs in the Creb3l1 promoter region (Figure 4A). These included NurRE half sites and NBRE forward and reverse motifs (Figure 4B). We first validated the 4.9 kb luciferase reporter construct by treating transfected cells with FSK, dexamethasone and p38 inhibitor (Figure 4C). Treatment of cells with FSK robustly increased luciferase activity in both cell lines consistent with activation of Creb3l1 expression by cAMP pathways. FSK-induced increases in Creb3l1 promoter activity were attenuated by dexamethasone. However, treatment with the p38 inhibitor SB202190 decreased promoter activity only in AtT20 cells.

To identify Nr4a1 interaction sites in the Creb3l1 promoter-luciferase assays were performed on a series of promoter deletion constructs. Overexpression of Nr4a1 as well as FSK treatment robustly increased activity of the 4.9 kb promoter construct in both cell-types (Figure 4D). Truncation of the Creb3l1 promoter from 4.9 kb–3 kb greatly diminished promotor activation by FSK and Nr4a1, more so in AtT20 cells than N2a cells. Thus, indicating that the primary site for activation by cAMP pathways and Nr4a1 is located within this 1.9 kb segment of DNA which contains NBRE and NurRE responsive elements. Further deletions by restriction digests of the promoter region narrowed the search to a 0.6 kb section of DNA situated between 3.0 kb and 3.6 kb, which contains a single NBRE site, NBRE3 (Figure 4E). Accordingly, specific deletion mutants for NBRE3 and also NBRE2 were generated. Deletion of NBRE3, but not NBRE2, profoundly attenuated FSK and Nr4a1 activation of the Creb3l1 promoter in AtT20 and N2a cells (Figure 4F). In AtT20 cells deletion of NBRE3 completely abolished activation of the Creb3l1 promoter by Nr4a1 showing that this site is important for the actions of Nr4a1.

**Figure 5:**
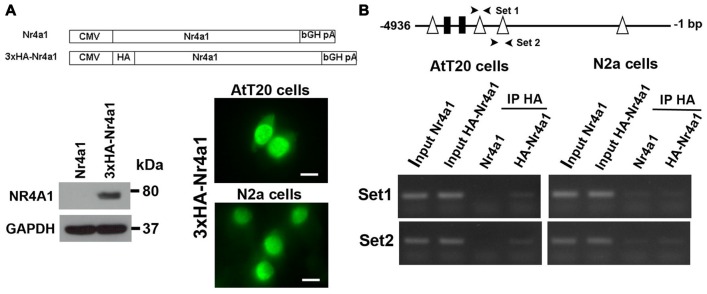
Nr4a1 regulates Creb3l1 transcription by binding at the promoter. **(A)** Validation of HA tagged Nr4a1 construct by immunoblot in AtT20 cells and immunofluorescence in AtT20 and N2a cells. **(B)** ChIP assays were performed using antibody against HA tag in Nr4a1 and HA-Nr4a1 transfected cells. Two sets of primers (arrow heads) were designed to amplify promoter regions close to NBRE2 and NBRE3 consensus sequences by RT-PCR. PCR products from the ChIP assay were verified by gel electrophoresis. Scale bars = 10 μm.

To demonstrate direct binding of Nr4a1 to this promoter region we performed ChIP assays in AtT20 and N2a cells. We made a 3×HA tagged NR4a1 construct and verified its expression by Western blot in AtT20 cells and cellular localization of the expressed protein by immunofluorescence in both AtT20 and N2a cells (Figure 5A). The strong localization of Nr4a1 protein in the nucleus is consistent with it having a role as a transcriptional regulator. In AtT20 cells, the Nr4a1 binding DNA fragments were identified by PCR using specific mouse Creb3l1 promoter primers. The correctly sized bands were enriched in anti-HA tag immunopreciptated DNA from the HA-Nr4a1 but not Nr4a1 transfected AtT20 cells, suggesting binding of Nr4a1 to the Creb3l1 promoter in this region (Figure 5B). There was no enrichment of immunopreciptated DNA in ChIP experiments performed in N2a cells, suggesting little or no binding to the Creb3l1 promoter in this cell line.

### Epigenetic Silencing of the Creb3l Gene by CpG Promoter Methylation Inhibits Activation by Nr4a1

**Figure 6:**
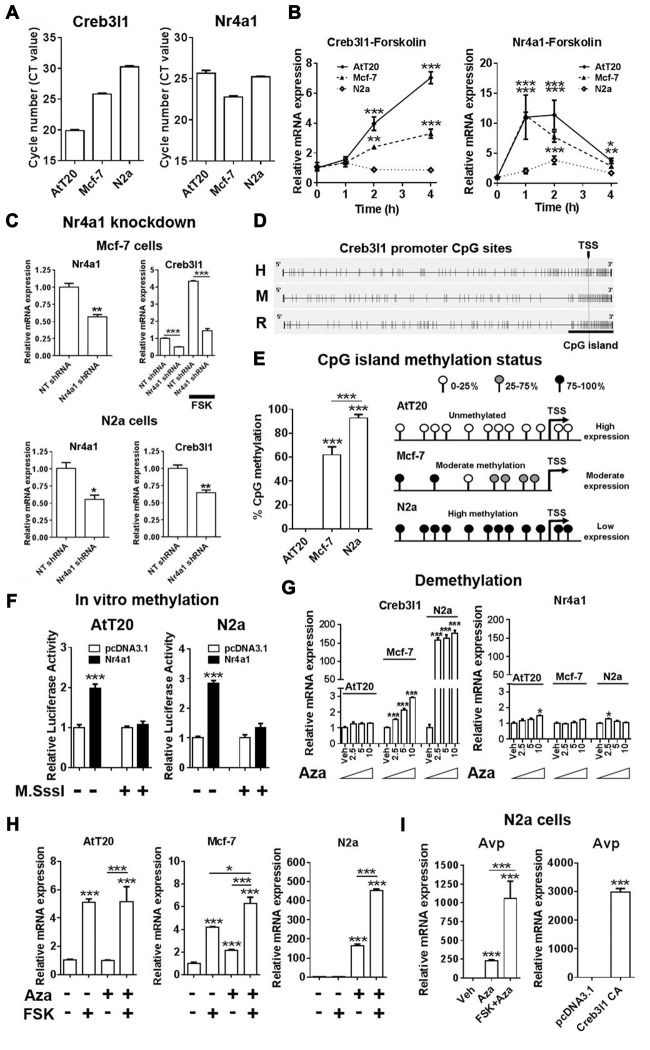
Epigenetic silencing of the Creb3l1 gene by CpG promoter methylation. **(A)** Expression of Creb3l1 and Nr4a1 in different mammalian cell lines presented as CT value. **(B)** Time course of FSK treatment in three different cell lines. **(C)** The effect of Nr4a1 knockdown on Creb3l1 expression in Mcf-7 and N2a cells. **(D)** Identification of a conserved CpG island in the human and rodent Creb3l1 promoters (4.9 kb). **(E)** Methylation status of the Creb3l1 promoter CpG island in three different cell lines. **(F)** Luciferase assays of methylated Creb3l1 luciferase reporter constructs with overexpression of Nr4a1 or 4 h FSK treatment. **(G)** qRT-PCR analysis of Creb3l1 and Nr4a1 in cell lines treated with doses of 2.5–10 μM of 5-Aza-2′deoxycytidine for 72 h. **(H)** FSK stimulation (4 h) of cells treated with 5-Aza-2′deoxycytidine (72 h, 5 μM). **(I)** The effect of 5-Aza-2′deoxycytidine and FSK treatment on expression of arginine vasopressin (Avp) in N2a cells. Increase of Avp mRNA expression by overexpression of a constitutively active (CA) mutant of Creb3l1 in N2a cells. TSS, transcription start site. Values are means + SEM of *n* = 3−4 per group. *p < 0.05, **p < 0.01, ***p < 0.001.

We next investigated Nr4a1 and Creb3l1 mRNA expression in three different cell lines, AtT20, N2a and Mcf-7, to better understand the relationship between the expression of these genes. We chose Mcf-7 cells because they have previously been reported to express Creb3l1 (Denard et al., 2012). Our data showed high Creb3l1 expression in AtT20 cells, moderate expression in Mcf-7 and low expression in N2a cells (Figure 6A). In contrast, Nr4a1 was expressed at near equivalent levels in in all three cell lines, suggesting factors other than Nr4a1 were influencing the expression of Creb3l1. Treatment with FSK increased Nr4a1 expression in all three cell lines, but Creb3l1 expression increased in only AtT20 and Mcf-7 cells (Figure 6B). Knockdown of Nr4a1 expression by lentiviral delivery of specific shRNAs targeting Nr4a1 decreased Creb3l1 expression in Mcf-7 and N2a cells, and blunted the increase in Creb3l1 abundance in Mcf-7 cells treated with FSK, similar to our earlier findings in AtT20 cells (Figure 6C). We asked if methylation of the Creb3l1 promoter could be responsible. Using Methyl Primer Express Software we identified a single CpG island in the proximal promoter/5′UTR region of rodent and human Creb3l1 (Figure 6D). Analysis of individual CpGs in the proximal promoter region showed that this region was unmethylated in AtT20 cells, moderately methylated in Mcf-7 cells and highly methylated in N2a cells, consistent with high, moderate and low expression of Creb3l1 mRNA in the respective cell lines (Figure 6E). To see if methylation of the Creb3l1 promoter could affect activation of Creb3l1 transcription specifically by Nr4a1, we performed *in vitro* methylation studies in AtT20 and N2a cells. *In vitro* methylation of Creb3l1 promoter-luciferase constructs by CpG methyltransferase blocked activation of the Creb3l1 promoter by overexpression of Nr4a1 (Figure 6F). In contrast, demethylation of DNA by treatment with 5-Aza-2′deoxycytidine dose dependently increased endogenous Creb3l1 expression in Mcf-7 cells and dramatically increased Creb3l1 expression in N2a cells (Figure 6G). Moreover, treatment of AtT20 cells with this chemical had no effect on Creb3l1 expression consist with the absence of methylation marks on the Creb3l1 promoter in this cell line. The expression of Nr4a1 was largely unaltered by these treatments. To see if we could rescue cAMP inducibility of Creb3l1, we treated cells with FSK in addition to 5-Aza-2′deoxycytidine. Treatment of Mcf-7 and N2a cells with FSK was able to further upregulate Creb3l1 expression (Figure 6H). Therefore, methylation of the Creb3l1 promoter dictates Creb3l1 mRNA abundance in these cell lines where Nr4a1 is similarly expressed.

As Creb3l1 is known to be a putative transcription factor of the Avp gene (Greenwood et al., 2014), we tested if demethylation could induce Avp expression in N2a cells (Figure 6I). Treatment with Aza alone increased Avp expression, and when combined with FSK this increase in expression was markedly enhanced. To demonstrate that increased Avp expression was not the result of demethylation of the Avp promoter as we have previously reported (Greenwood et al., 2016a), we overexpressed Creb3l1 in N2a cells and were able to successfully reproduce this response. Therefore, methylation strongly influences the actions of Nr4a1 as a transcriptional regulator of the Creb3l1 gene *in vitro*.

### Transcriptional Regulation of Creb3l1 Expression in the Rat PVN

**Figure 7:**
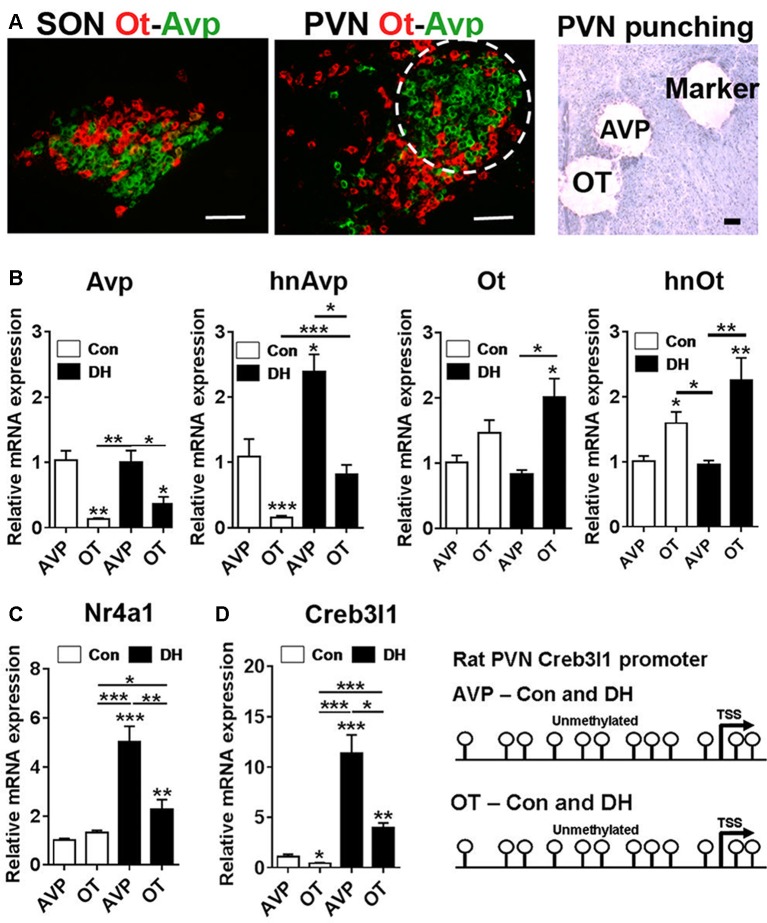
Transcriptional regulation of Creb3l1 expression in the rat PVN. **(A)** Fluorescent *in situ* hybridization marking the distribution of Avp and Ot mRNAs in the SON and PVN. The toluidine blue stained section illustrates the punches collected for mRNA analysis. In the PVN the enrichment of AVP positive neurones in the posterior magnocellular bundle (labeled AVP) and OT positive neurones more medially (labeled OT) enabled a level of separation of these subdivisions. A 27 gauge needle was then used to create small tracts lateral to the posterior magnocellular subdivision of the PVN (labeled marker) for orientation of punching. **(B)** qRT-PCR analysis of Avp and Ot RNAs in AVP and OT punches in the euhydrated and 3 days dehydrated rat PVN. **(C)** qRT-PCR analysis of Nr4a1 and Creb3l1 expression in AVP and OT punches in the euhydrated and 3 days dehydrated rat PVN. **(D)** Methylation status of 12 CpGs on the Creb3l1 promoter in DNA extracted from AVP and OT punch samples of euhydrated and dehydrated rat. DH, dehydration. Values are means + SEM of *n* = 4 per group. *p < 0.05, **p < 0.01, ***p < 0.001. Scale bars = 100 μm.

To place our *in vitro* cellular findings within the context of the whole organism, we looked at the molecular mechanisms that alter Creb3l1 expression in the PVN of control and 3 days dehydrated rats. We performed RNAscope to look at the distribution of Avp and Ot mRNAs in the PVN and SON of the rat hypothalamus (Figure 7A). The architecture of the PVN enables the enrichment of AVP neurones using a circular micropunch to collect AVP and OT enriched cellular populations. We show using qPCR that this technique is able to enrich for AVP and OT expressing cells in the PVN of control and 3 days dehydrated rats (Figure 7B). We then asked about Nr4a1 and Creb3l1 expression in these samples. There was no difference in Nr4a1 expression in the basal euhydrated condition. However, in response to DH, Nr4a1 expression was significantly increased in AVP compared to OT punch samples (Figure 7C). In agreement with the concept of Nr4a1 activating Creb3l1 transcription, Creb3l1 expression was also higher in AVP compared to OT punches in dehydrated rats. We next asked if methylation of the Creb3l1 promoter could explain the difference in Creb3l1 expression in AVP and OT punch samples in basal and dehydrated states (Figure 7D). We show the rat Creb3l1 promoter to be completely unmethylated in all samples tested. Therefore, the absence of methylation marks on the Creb3l1 promoter in the PVN allows for activation by transcription factors such as Nr4a1 within this brain nuclei.

## Discussion

ER stress has been at the forefront of investigations surrounding regulation of Creb3l1 synthesis and cleavage by RIP since this mechanism was first identified in C6 glioma cells (Murakami et al., 2006). Here, we show that Creb3l1 cleavage is constitutively active (CA) in AtT20 cells, with increased Creb3l1 transcription automatically leading to a greater abundance of N-terminal cleaved Creb3l1. Hence, understanding the pathways regulating the rate-limiting step of Creb3l1 transcription are pivotal to understand the cellular mechanisms activating Creb3l1. Here we identify orphan nuclear receptor Nr4a1 as a transcription factor of the Creb3l1 gene in AtT20 cells and also demonstrate that methylation is a major factor determining Creb3l1 expression *in vitro*. We show that Creb3l1 transcription is activated by this novel pathway *in vitro* and present compelling evidence that this mechanism may regulate Creb3l1 expression in AVP neurones of the rat hypothalamus in conditions of osmotic stress.

When stimulated by FSK, both full-length and cleaved forms of Creb3l1 protein rapidly accumulate in AtT20 cells. The proportion of inactive to active forms of Creb3l1 remains constant regardless of overall protein abundance, suggesting that cleavage by S1P and S2P is not a rate-limiting step in this process. Thus we proposed that transcriptional control of Creb3l1 by cAMP pathways is a rate-limiting step in the generation of active N-terminal Creb3l1. Two candidate pathways were identified for regulation of cAMP induced Creb3l1 expression, one inhibitory pathway regulated by the glucocorticoid dexamethasone, and one stimulatory pathway involving p38 MAP kinase. We have previously shown that the corticosteroid medication dexamethasone inhibits basal and FSK-induced Creb3l1 expression in AtT20 cells (Delidaki et al., 2011; Greenwood et al., 2015a). Here we show that basal and FSK-induced expression of Creb3l1 are also regulated by the p38 MAPK kinase pathway in this cell line. Inhibition of Creb3l1 expression by p38 inhibitors has been reported in human placental cell-line BeWo suggesting that this signaling pathway is not exclusive to our model (Delidaki et al., 2011).

We show that stimulation of Creb3l1 expression by FSK is blunted in the presence of protein synthesis inhibitor CHX. The need for *de novo* protein synthesis for cAMP stimulation of Creb3l1 expression implied that intermediary factor/s, also activated by cAMP pathways, are necessary for the initiation of Creb3l1 transcription. By systematically eliminating candidate transcriptional regulators of Creb3l1 sourced from microarray data of the SON (Hindmarch et al., 2006), we identified orphan nuclear receptor Nr4a1 as being important for Creb3l1 transcription.

Nr4a1 is classified as an orphan because no endogenous ligand has yet been identified. This receptor behaves as an immediate early gene, and as such, its mRNA is induced rapidly in response to a stimulus, a process that occurs independently of protein synthesis (Maruyama et al., 1995). The expression of Nr4a1 is well-known for being activated by cAMP pathways (Maruyama et al., 1995). Moreover, the regulation of Nr4a1 expression has also been shown to involve the activation of p38 (Li et al., 2015; Shao et al., 2016). The subcellular localization of Nr4a1 is important for its function, and as such, an increase in Nr4a1 expression is not necessary to increase its activity, which can be achieved by posttranslational modifications such as phosphorylation (Fahrner et al., 1990). In the nucleus, Nr4a1 can act as a transcriptional modulator by binding as a monomer, homodimer and heterodimer at specific DNA responsive elements such as NurRE and NBRE (Philips et al., 1997). We provide strong evidence that Nr4a1 interacts with the Creb3l1 promoter at single NBRE site in its promoter to regulate Creb3l1 transcription.

Nr4a1 has been reported to bind and activate transcription of NurRE and NBRE reporter constructs, without requiring any additional factors (Davis et al., 1991; Wilson et al., 1991). This said, members the Nr4a receptor family have been shown to interact with each other and a variety of co-regulators for optimal gene regulation (Campos-Melo et al., 2013). Nr4a1 and Nr4a2 can form heterodimers that interact with NurRE sequences to enhance transcription of reporters (Maira et al., 1999). Furthermore, Nr4a1 has also been shown to form heterodimers with the retinoid × receptor, receptors of 9-cis retinoic acid, which have roles in multiple nuclear receptor signaling pathways (Morita et al., 2005). Another binding partner of Nr4a1 is KRAB domain association protein 1 (Kap1), which was first identified as a co-activator of Nr4a1 transcription in AtT20 cells (Rambaud et al., 2009). Nr4a2, Kap1 and retinoid × receptors are expressed in the hypothalamus (Kawasaki et al., 2005; Rambaud et al., 2009; Johnson et al., 2015). Therefore, these Nr4a1 transcription factor complexes may be important in the regulation of Creb3l1 expression, although this remains to be established.

The identification of Nr4a1 as a transcription factor of the Creb3l1 gene may have importance in cancer research. The expression levels of Nr4a1 and Creb3l1 have been used a biomarkers to grade cancerous cells/tumors (Alexopoulou et al., 2010; Rose et al., 2014; Safe et al., 2014; Denard et al., 2015; Ward et al., 2016). No connection has currently been made between Nr4a1 and Creb3l1 expression in cancer cells, but there are studies that support this concept. A study by Mellor et al. (2013) reported low Creb3l1 expression in metastatic breast cancer cells lines compared to cell lines that had little or no metastatic capabilities (Mellor et al., 2013). A separate study investigating Nr4a1 expression in breast tumors similarly reported low Nr4a1 expression in higher grade and metastatic breast tumors (Alexopoulou et al., 2010). In some cases, studies have shown that aggressive phenotypes of breast and bladder cancer have low Creb3l1 expression resulting from Creb3l1 promoter hypermethylation (Rose et al., 2014; Ward et al., 2016). This is consistent with our findings in breast cancer cell line Mcf-7 and in N2a cells. Our data now supports the investigation of both Nr4a1 and Creb3l1 in cancer studies. The recent development of Nr4a1 agonists that directly bind and activate Nr4a1 may be useful pharmacological tools to increase Creb3l1 expression in tumor cells (Cho et al., 2007; Liu et al., 2010), but our data shows that this would certainly depend on the methylation status of the Creb3l1 promoter.

We placed our *in vitro* findings of Creb3l1 regulation from our study of AtT20 cells into an *in vitro* systems context. For many years, studies have associated the expression of Nr4a1 with activation of Avp, Ot and corticotropin releasing hormone transcription in the PVN and SON by cAMP pathways (Chan et al., 1993; Kawasaki et al., 2005; Girotti et al., 2007). The spatial pattern of Nr4a1 mRNA distribution in the PVN is dependent on the nature of the stimulus with restraint stress strongly inducing Nr4a1 expression in parvocellular divisions of the PVN (Imaki et al., 1996), while in DH and SL expression is largely detected in magnocellular divisions of PVN and SON (Chan et al., 1993).

Our data agrees with the previous concept of activation of Avp transcription *in vitro* by Nr4a1, but suggests that this mechanism requires the intermediary step of transcriptional activation of the Creb3l1 gene to promote Avp transcription. In addition, our findings from *in vitro* cell cultures hinted that methylation could be important in regulating Creb3l1 expression in different cell-types of the PVN, namely AVP and OT neurones, particularly as Creb3l1 expression increases exclusively in AVP neurones by DH (Greenwood et al., 2014). We recently described altered methylation patterns of the Avp promoter with DH in the SON and in this study we observed decreased expression of the demethylating enzyme ten-eleven translocation 2, Tet2, by DH and SL in support of methylation changes in the hypothalamus (Greenwood et al., 2016a). Interestingly there was an absence of methylation marks on this region of the Creb3l1 promoter *in vitro* in the PVN. This suggests that the differences in Creb3l1 expression in AVP and OT neurones in states of DH is achieved through changes to the transcriptional complement within these two distinctive cell-types rather than any alterations to promoter methylation. This is consistent with the strong up-regulation of Nr4a1 and Creb3l1 in AVP magnocellular neurones by 3 days of DH in the current study.

**Figure 8:**
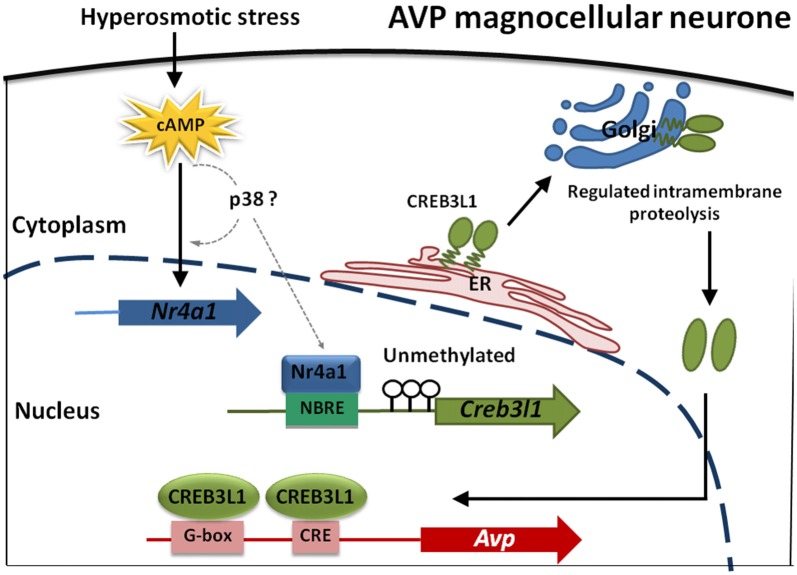
Modeling transcriptional control of the Avp gene in magnocellular neurones of the hypothalamus. cAMP levels increase in magnocellular neurons in response to raised plasma osmolality resulting from DH. Our *in vitro* studies have indicated that p38 may increase Nr4a1 and also Creb3l1 mRNA expression but any direct actions *in vitro* and in magnocellular neurones remain to be established (dotted lines). What is known is that Nr4a1 expression increases in AVP magnocellular neurons alongside increased expression of Creb3l1 in the dehydrated rat. Due to an absence of methylation marks on the Creb3l1 promoter *in vivo*, Nr4a1 has the capacity to increase Creb3l1 transcription through it interactions with a single NBRE site in the promoter resulting in increased full-length Creb3l1 protein. Inactive Creb3l1 protein is anchored in the endoplasmic reticulum (ER) membrane. Upon stimulation by DH, Creb3l1 is transported to Golgi apparatus where it is activated by regulated intramembrane proteolysis (RIP). The N-terminal active form of Creb3l1 then enters the nucleus to activate transcription of Avp.

In summary, our data shows that transcription of Creb3l1 is a primary factor influencing the availability of N-terminal active Creb3l1, and is thus the rate-limiting step in activating transcriptional targets of this gene. One of the factors regulating Creb3l1 expression is the nuclear receptor Nr4a1, which for many years has been postulated to be one of the key players in the transcriptional cascade that is activated in the hypothalamus in conditions of raised plasma osmolality. We show that the abundance/activity of Nr4a1 is not the limiting factor in increasing the abundance of Creb3l1 but that this is dictated by the methylation status of the Creb3l promoter region in our *in vitro* studies. Our experiments have culminated in the identification of a novel cAMP-Nr4a1-Creb3l1 transcriptional pathway. One of the outputs of this pathway will be increased Avp transcription in magnocellular neurones of the hypothalamus (Figure 8). Taken together, this study provides a novel mechanism of Creb3l1 activation by Nr4a1 that adds important understanding to transcriptional mechanisms not only in the hypothalamus but also possibly more broadly within cancer research.

## Author Contributions

MPG and DM conceived the study. MPG and MG designed, performed and analyzed experiments and wrote the article. BTG and RCD designed and performed experiments.

## Conflict of Interest Statement

The authors declare that the research was conducted in the absence of any commercial or financial relationships that could be construed as a potential conflict of interest.
